# Differential Actions of Chlorhexidine on the Cell Wall of *Bacillus subtilis* and *Escherichia coli*

**DOI:** 10.1371/journal.pone.0036659

**Published:** 2012-05-11

**Authors:** Hon-Yeung Cheung, Matthew Man-Kin Wong, Sau-Ha Cheung, Longman Yimin Liang, Yun-Wah Lam, Sung-Kay Chiu

**Affiliations:** Research Group for Bioactive Products, Department of Biology & Chemistry, City University of Hong Kong, Hong Kong SAR, People's Republic of China; Institut Pasteur, France

## Abstract

Chlorhexidine is a chlorinated phenolic disinfectant used commonly in mouthwash for its action against bacteria. However, a comparative study of the action of chlorhexidine on the cell morphology of Gram-positive and Gram-negative bacteria is lacking. In this study, the actions of chlorhexidine on the cell morphology were identified with the aids of electron microscopy. After exposure to chlorhexidine, numerous spots of indentation on the cell wall were found in both *Bacillus subtilis* and *Escherichia coli.* The number of indentation spots increased with time of incubation and increasing chlorhexidine concentration. Interestingly, the dented spots found in *B. subtilis* appeared mainly at the hemispherical caps of the cells, while in *E. coli* the dented spots were found all over the cells. After being exposed to chlorhexidine for a prolonged period, leakage of cellular contents and subsequent ghost cells were observed, especially from *B subtilis*. By using 2-D gel/MS-MS analysis, five proteins related to purine nucleoside interconversion and metabolism were preferentially induced in the cell wall of *E. coli*, while three proteins related to stress response and four others in amino acid biosynthesis were up-regulated in the cell wall materials of *B. subtilis*. The localized morphological damages together with the biochemical and protein analysis of the chlorhexidine-treated cells suggest that chlorhexidine may act on the differentially distributed lipids in the cell membranes/wall of *B. subtilis* and *E. coli*.

## Introduction

Chlorhexidine (CHX) is one of the most favorable choices amongst all the disinfectants currently in use. It has been reported that CHX is effective to prevent and control infectious diseases of the mouth by killing bacteria in saliva and tongue [1]; yet it has little undesirable effects [2]. In addition, it is a broad-spectrum bactericidal agent against both Gram (+) and (−) bacteria and fungi [3], [4]. Given that CHX has a strong antimicrobial activity but relatively low levels of toxicity to mammalian cells [5], [6], it is regarded as the most useful and safe disinfectant. Its propensity to bind to the surface of tissues offers a long-lasting antimicrobial effect [7]. This property of CHX also makes it suitable for use as a preservative in some pharmaceutical or medical products, such as ophthalmic solutions, and as a disinfectant of medical instruments and hard surfaces.

The effect of CHX on bacteria has been studied extensively in recent years. These include investigation on its efficacy in cleansing and disinfection program [7], prevention of periodontal diseases [8] and others. Though the *in vivo* and *in vitro* antimicrobial activities of CHX have been reported, the exact mechanism of action exerted by CHX on bacteria and the differences in activity on Gram-positive and -negative bacteria are still not very clear. It is generally thought that the cationic CHX interacts with the anionic phosphate residue of the lipid molecules in the cell membrane by adsorption. It has been postulated that CHX bypasses the cell wall exclusion mechanism, perfuses to cytoplasmic membrane to cause leakage of low molecular weight components through cell membrane, and precipitates cytoplasm content through the formation of complexes with phosphate moieties [9], [10]. And yet, no distinction has been made on its action between Gram-positive and Gram-negative bacteria.

In this study, we found that CHX induced the formation of dented spots on the cell wall of both Gram-positive and Gram-negative bacteria. The dented spots were found to be localized more on the polar region in *B. subtilis*, whereas in *E. coli*, the spots were evenly distributed along the cell body. CHX breached the cell walls of the bacteria and led to leakage of their cellular contents.

## Materials and Methods

### 1. Bacterial strains, Chemicals, and biocide solutions

*E. coli* ATCC 10536 and *B. subtilis* 60015 were used throughout this study. Chlorhexidine (CHX) and hexamethyldisilazane (HMDS) were purchased from Sigma-Aldrich (Saint Louis, Missouri, USA). Stock solution of CHX (75 mg/L) was prepared by dissolving in absolute ethanol. Nutrient agar and nutrient broth were obtained from Oxoid Chemical Company (Basingstoke, Hampshire, UK). Glutaraldehyde (Electron Microscopy Grade) was from Mallinckrodt Specialty Chemicals Company (Paris, KY). Osmium tetroxide, Spur's resin, uranyl acetate and lead citrate were purchased from Electron Microscopy Sciences (Hatfield, Pennsylvania, USA).

### 2. Growth study

Growth of the bacterial cultures was monitored by measuring OD_600_ in shaker water bath (115 rpm) at 37°C. Overnight culture was harvested and re-suspended in an aliquot of fresh nutrient broth before it was inoculated into 25 ml of the medium containing different concentrations of CHX. Culture was grown aerobically at 37°C by vigorous shaking and was measured at OD_600_ at 1 h interval over a 24 h period. The growth conditions for other experiments were the same as described above.

### 3. Determination of the minimal inhibitory concentration (MIC) of chlorhexidine on bacteria

MIC value of chlorhexidine was determined according to the procedures recommended by the National Committee for Clinical Laboratory Standard (NCCLS) or CLSI. In brief, chlorhexidine at 0.1, 0.2, 0.3, 0.4, 0.5, and 0.6 mg/L were added to nutrient broth and 1% of ethanol as the solvent of chlorhexidine was added to each culture. Bacteria of both species were incubated at 37^o^C and OD_600_ readings of each culture were measured continuously for 4 hours. The lowest concentration of chlorhexidine that gave the optical density reading of 0.01 was the MIC of that bacterial culture.

### 4. Environmental scanning electron microscope (ESEM) study

The procedure of SEM was performed as described by Khunkitti et al. [11] with some modifications. In most experiments, bacterial cells treated with 0.75 mg/L of CHX were fixed for 24 h at 4°C in 2% v/v glutaraldehyde in 0.1 M sodium cacodylate buffer, pH 7.2. To test the effect of different concentrations of CHX on the cells, 0 mg/L, 0.3 mg/L, and 0.75 mg/L of CHX were used. The cells were washed with 0.1 M, 0.05 M and 0.025 M of cacodylate buffers for 15 min each. Samples were then dehydrated through a graded ethanol series of 50, 70 and 90% v/v for 15 min each, washed thrice with 100% ethanol for 15 min each and with hexamethyldisilazane (HMDS) twice for 30 s each. With the use of carbon tape, the samples were stuck on aluminium stubs and kept in desiccators overnight. The dehydrated samples were observed under a Philips XL30 ESEM-FEG environmental scanning electron microscope (Philips Electronics, Netherlands, Europe) after coated with carbon.

### 5. Determination of cellular content of chlorine and phosphorus by energy dispersive X-ray analysis (EDAX)

Elemental content of chlorine and phosphorus in individual cells was determined by an energy-dispersive X-ray detector equipped with a multichannel analyzer (Link 860 EDAX) associated with the ESEM. The microscope was operated with accelerating voltages in the range of 15–20 KV and the magnification was between 15,000X and 30,000X. The cellular content of chlorine and phosphorus in the bacterial specimen encircled within an area was recorded and then output as an EDAX spectrum. The percentage composition of the detected elements was calculated. Weight percentages of chlorine and phosphorus reflect the cellular content of CHX and phospholipids in the cells, respectively [12]–[14].

### 6. Transmission electron microscopy (TEM) studies

TEM was performed as described previously [15] with some modifications. Bacterial cells exposed to 0.75 mg/L of CHX for 4 h were harvested and fixed with 3% v/v glutaraldehyde solution at 4°C overnight. Thin sections of the bacterial samples were prepared as described and photographed using a Philips Tecnai 12 transmission electron microscope (Philips Electronics, Netherlands, Europe) operating at 80 kV with a liquid-nitrogen cold trap in place.

### 7. Counting of dented spots

**Figure 1:**
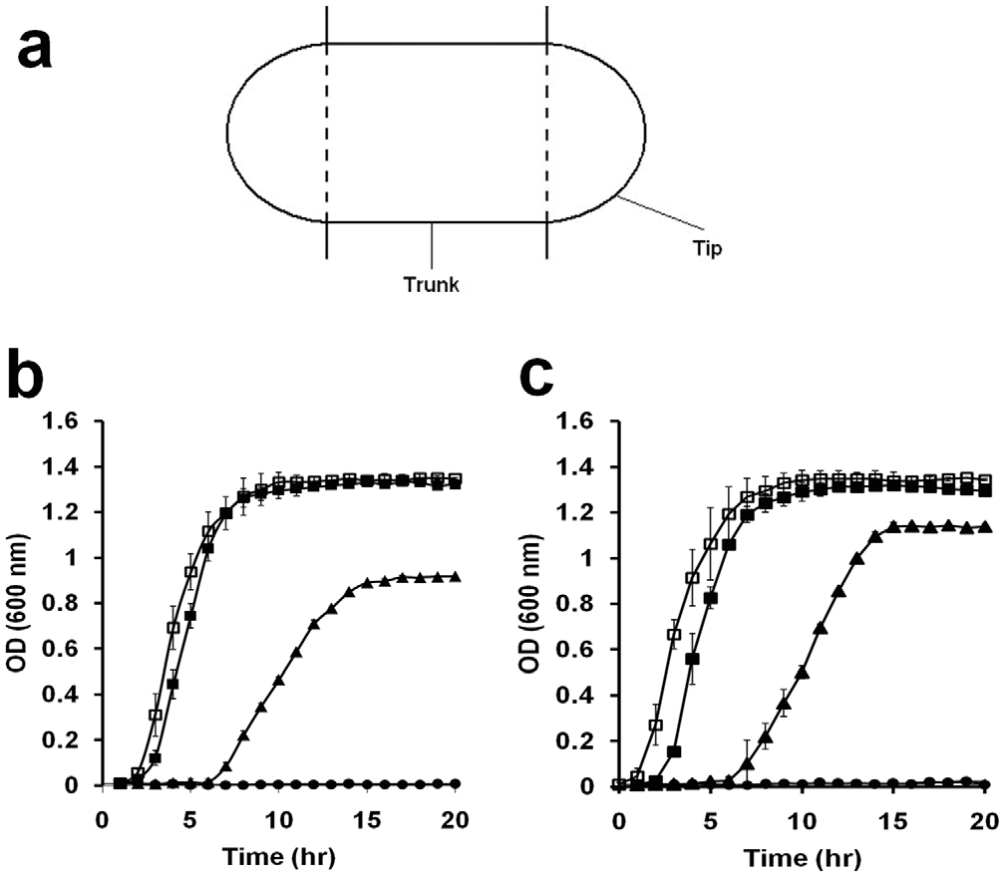
Effect of chlorhexidine on the growth of bacterial cells. (a) Definition of trunk and tip of a bacterial cell. The two hemispherical caps are the tips while the middle cylindrical zone is the trunk of a rod-shaped bacterial cell as described in Materials and methods. (b and c) The growth of *B. subtilis* (b) and *E. coli* (c) was monitored by measuring the optical density of the culture. The culture was sampled at a particular time point and OD at 600 nm was measured. The concentrations of chlorhexidine added to the culture were: 0 (open rectangle), 0.25 (filled rectangle), 0.5 (closed triangle), 0.75 (closed circle) mg/L. Each plot of the curve is the average of three readings and standard deviation is shown.

With the aid of the environmental scanning electron microscope, the number of dented spots at different parts (trunk and tip) in 50 cells of *B. subtilis* and *E. coli* was counted. The tip is defined as the hemispherical caps of a rod-shaped bacterial cell while the trunk is defined as the remaining part (cylindrical zone) of the bacterial cell (Fig. 1a).

### 8. SDS polyacrylamide gel analysis of total protein from the CHX treated bacteria

Both *E. coli* and *B. subtilis* cells from 250 ml cultures of nutrient broth at 0.35 OD_600_ were collected, rinse twice with phosphate buffered saline (PBS), re-suspended and cultured further in pre-warmed 250 ml nutrient broth as a control or nutrient broth with 0.75 mg/L CHX at 37°C, 115 rpm. Samples after incubating for 0, 10, 30, 120, and 240 min. were collected and rinsed twice with PBS before lysed directly with laemmli buffer at 95°C for 10 minutes. Equivalent number of bacteria according to the OD measurement of each sample was loaded into a 10% SDS-polyacrylamide gel.

### 9. Identification of proteins from two-dimensional gel electrophoresis and mass spectrometry

One litre of log phase cultures of both *E. coli* and *B. subtilis* were spun down and rinsed twice with PBS. First half of the culture was re-suspended in PBS. The second half was re-suspended in nutrient broth containing CHX and incubated at 37°C for 4 hours. The cell suspension in PBS was lysed using a Mikro-dismembrator II (B. Braun, Germany) with glass beads until the majority of the cells were ruptured as examined under high power light microscope. The lysate was then centrifuged at 6,000× *g* for 12 min to remove unbroken cells. The supernatant of the lysate was then centrifuged at 25,000× *g* for 1 hour to pellet the wall/membrane fraction, which was cleaned further by rinsing twice with PBS. The pellet was solublized in 2D lysis buffer (8 M urea, 4% CHAPS, 2% Pharmalyte, 40 mM DTT and 1 mM PMSF). Acetone precipitation and 2D gel separation of the solubilized cell wall/membrane fraction were performed as per the supplier's instruction (GE healthcare, UK). Approximately 150 μg of the protein extracted from each sample was loaded onto a 7 cm IEF strip (pH 3–10) and subjected to isoelectric focussing, followed by placing the strip on a 12% acrylamide gel for the second dimension.

Afterward the gels were fixed in a solution of 50% methanol and 10% acetic acid and stained with Coomassie Brilliant Blue. Image of the gel was taken by an LAS-4000 (Fujifilm, USA). Spot detection, matching, and data analysis were performed with Progenesis SameSpots (Nonlinear Dynamics, North Carolina, USA). Individual gel spots were destained with 1∶1 mixture of 50 mM NH_4_CO_3_/acetonitrile, dehydrated with acetonitrile and dried down in vacuum centrifuge. In-gel digestion of protein was performed by incubation overnight at 37°C in 20 μl of 1.25 ng/μl of trypsin solution (in 25 mM NH_4_CO_3_). Peptides were extracted twice in a solution of 50% acetonitrile and 1% formic acid with sonication for 10 minutes. The resulting solution was dried in vacuum centrifuge and dissolved in 5 μl of 0.1% formic acid.

One microliter of the peptide solution was spotted manually onto an ABI MALDI target plate and the spots were allowed to dry. One microliter of saturated matrix solution (α-cyano-4-hydroxycinnamic acid (CHCA) dissolved in 70% v/v acetonitrile) was then added to each well of the target plate, air-dried, and analyzed with a 4700 Proteomics Analyzer (Applied Biosystems, Framingham, MA, USA). When acquiring MS spectra, laser intensity was set at 5400 and ions were collected between 900 and 3,000 Da. All the acquired MS spectra represented signal averaging of 2,000 laser shots. When carrying out the MS/MS acquisition, eight most intensive peptides with S/N exceeding 100 from each spot were selected and subjected to subsequent MS/MS analysis. Each MS/ MS spectrum was compiled from 2,500 shots with laser intensity set to 6400. The collision energy was set at 1 kV and the collision gas was air. All mass data were searched by ProteinPilot v.3.0 (with MASCOT as the database search engine) with peptide and fragment ion mass tolerance of 0.5 Da. The database used was the NCBI database (version 07–2010) and bacteria taxonomy was chosen with 6,124,310 sequences. Peptide differential modifications allowed during the search were carbamidomethylation of cysteines and oxidation of methionines. The maximum number of missed cleavages was set to 2 with trypsin as the protease. Only the identification results with confidence level more than 95% were selected as positive hits.

## Results

### Effect of CHX on the growth of *B. subtilis* and *E. coli*

Since ethanol was used as the solvent to prepare the stock CHX solution, the antimicrobial activity of ethanol itself in the stock solution had to be determined. The growth of *Bacillus subtilis* and *Escherichia coli* cells in culture broth containing different concentrations of ethanol was measured. It was found that their growth was not affected when the final concentration of ethanol was not more than 4% v/v (data not shown). To eliminate the antimicrobial effect of ethanol itself on the bacterial cells in subsequent experiments, 1% v/v ethanol was added to all subsequent bacterial culture. In the absence of any chlorhexidine, both *B. subtilis* and *E. coli* grew at the same rate (Figure 1b and c, open rectangles). The inhibitory effect of CHX on cell growth was enhanced with increasing CHX concentration. When the CHX concentration was 0.5 mg/L (closed triangles), significant inhibition on the growth of both *B. subtilis* (Fig. 1b) and *E. coli* (Fig. 1c) was observed. The inhibitory action of CHX lasted for 6 h before the cells regained their ability to grow. After that, both *B. subtilis* and *E. coli* grew at a similar rate for another 4 h, but *B. subtilis* entered the stationary phase at a lower cell density than *E. coli* (Figure 1b and c, closed triangles). This suggests that *B. subtilis* was just slightly more sensitive to chlorhexidine than *E. coli*. At 0.75 mg/L of chlorhexidine (Figure 1b and c, closed circles), both species of bacteria were not able to grow at all. Then, we determined the minimum inhibitory concentration (MIC) of chlorhexidine on these bacteria, which is the lowest concentration of a chemical required to inhibit or stop the growth of a culture. The MIC for *B. subtilis* was 0.6 mg/L and for *E. coli* was 0.7 mg/L at 24 h, respectively. All the above data suggests that *B. subtilis* was slightly more susceptible to CHX than *E. coli*.

### Uptake of chlorhexidine and loss of phosphorus from CHX-treated cells

**Figure 2:**
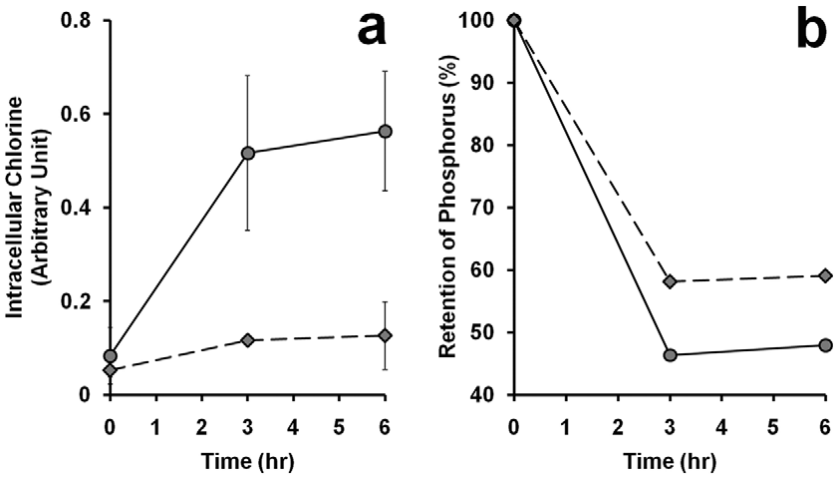
Uptake of chlorine and loss of phosphorus by bacterial cells in 0.75 **mg/L chlorhexidine.** The cellular contents of chlorine (a) and phosphorus (b) in the culture of *B. subtilis* (diamond) or *E. coli* (circle) at different time points were subjected to analysis using EDAX with an environmental scanning electron microscope (ESEM) as described in Materials and Methods. Standard errors were calculated based on the results from three separate samples. Experiment in (b) was repeated twice and the averages of the two were plotted.

Due to the cationic nature of chlorhexidine, it may bind to the negatively charged cell wall of bacteria and attack the cell membranes. The relative amount of CHX molecule appeared in the cells can be measured with an environmental scanning microscope using the energy dispersive X-ray (EDAX) analysis. Both the cellular contents of chlorine (Cl in CHX) and phosphorus (P) were measured after treating the bacterial cells with 0.75 mg/L chlorhexidine (Fig. 2). It was found that the amount of CHX in the cells increased with time of incubation and the uptake of CHX by *E. coli* (circles) was higher than that of *B. subtilis* (diamonds) at any time point (Fig. 2a). Coincidentally, the rate of loss of phosphorus from the cells was slightly faster in *E. coli* than *B. subtilis* in the first three hours after incubation with CHX (Fig. 2b).

### Morphological changes of bacteria after CHX treatment

**Figure 3:**
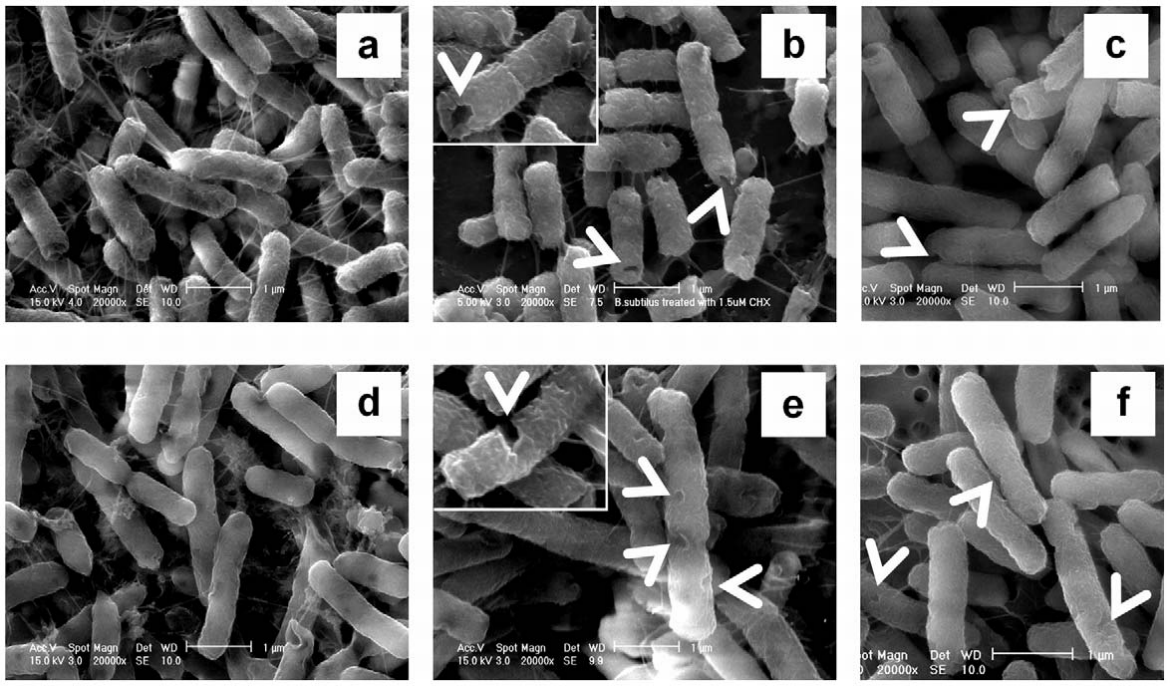
Morphological changes of *B. subtilis* and *E. coli* after exposure to chlorhexidine and acetone. The two species of bacterial cells exposed to 0.75 mg/L of chlorhexidine for 4 h were harvested and prepared as described in Materials and Methods. They were examined with an environmental scanning electron microscope after glutaraldehyde fixation. From (a) to (c) the cells were *B. subtilis* and from (d) to (f) the cells were *E. coli*. *B. subtilis* (a) and *E. coli* (d) cells were directly mounted for electron-microscopic examination without any CHX treatment. In (b) and (e), the cells were treated with 0.75 mg/L chlorhexidine for 4 h. For (c) and (f), the cells were treated with 5% and 7% acetone for 4 h, respectively. These results were confirmed in two independent experiments. Magnification in all electron micrographs was x 20,000. Bar  = 1 μm. Magnification of the inserts of (**b**) and (**e**) was x 30,000.

**Figure 4:**
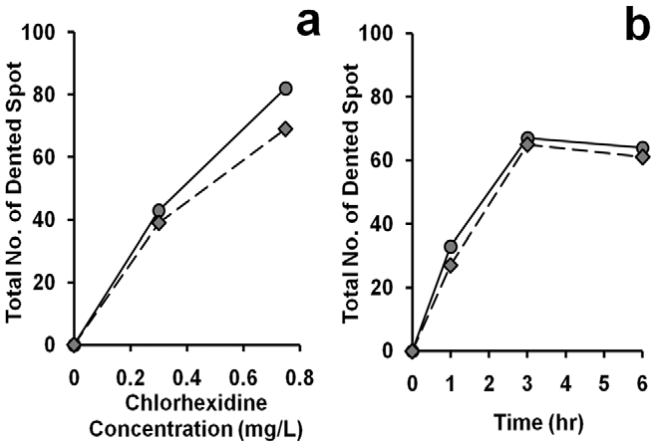
Total number of dented spot observed on cell surface. The dented spots on both the trunk and tips were counted in 50 cells of *B. subtilis* (**diamond**) and *E. coli* (**circle**) from cultures with (**a**) different chlorhexidine concentrations for 6 hr and (**b**) treated with 0.75 mg/L of chlorhexidine for different lengths of time. These results were confirmed in two independent experiments.

**Figure 5:**
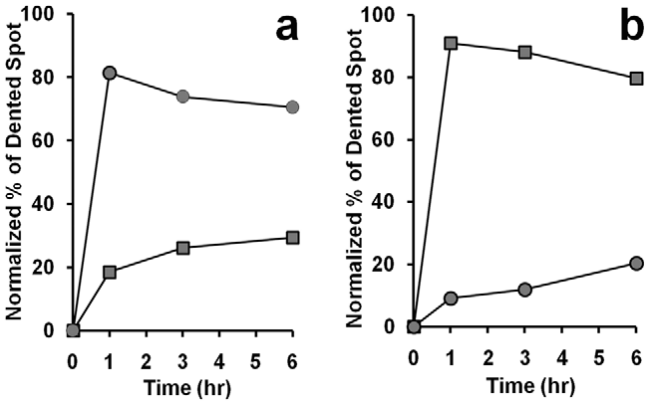
Time course of chlorhexidine treatment on the distribution of dented spots on the cell surface. Bacterial cells were incubated in media with 0.75 mg/L chlorhexidine over a 6 hr period. The bacterial cells at a particular time point were harvested and examined under an environmental scanning electron microscope (ESEM). The number of dented spots at the trunk (**rectangle**) and tip (**circle**) was counted in *B. subtilis* (**a**) and *E. coli* (**b**). Each time point represents the average number of dented spot on 50 cells. These results were confirmed in two independent experiments.

It has been known that CHX causes deformation in the cell wall of Gram-negative bacteria [16]. We were interested to investigate whether the effect of CHX on both Gram-positive and Gram-negative bacteria were similar, which may help us in understanding the mechanism of the anti-bactericidal effects on their cell wall structures. Without any CHX treatment, the cell wall surfaces of both *B. subtilis* and *E. coli* appeared smooth (Fig. 3a and 3d, respectively). Significant changes in cell morphology were observed in these bacteria after the treatment. Dented spots were found over the cell surface of *E. coli* including the tips and the trunk (Fig. 3e, white arrow head) while the indentation in *B. subtilis* was restricted mainly to the tip region of the cells (Fig. 3b, white arrow head). In both *E. coli* and *B. subtilis* the number of dented spots on cell surface increased with CHX concentration (Fig. 4a) and longer exposure to CHX led to more dented spots (Fig. 4b). Interestingly, we observed that most of the dented spots in *E. coli* were preferentially found in the trunk than the tips (Fig. 5b). In contrast, the dented spots on *B. subtilis* cells were located mainly at the tip regions (Fig. 5a). As argued in Discussion, we hypothesized that the action of chlorhexidine may be more specific on certain lipids in the cell membrane of the bacteria, and we also tested whether acetone, an organic solvent, might induce a similar change in the cell wall. Therefore, different concentrations of acetone were added to the cell culture and the cells were observed under ESEM. Dented spots were also observed in both *B. subtilis* and *E. coli* after exposing to acetone (Fig. 3c and 3f), although it required a slightly lower concentration of acetone to induce the formation of dented spots in *B. subtilis* cells (5%) than *E. coli* (7%).

**Figure 6:**
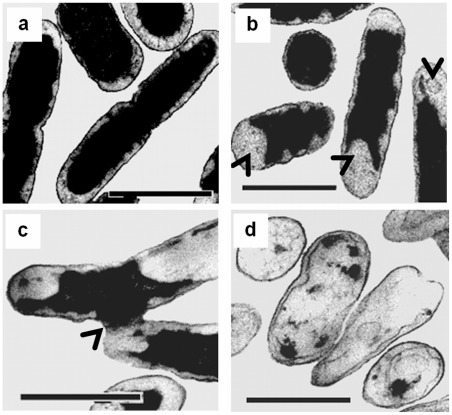
Cytological changes of *B. subtilis* after treatment with chlorhexidine. Control cells possess intact cell membrane, cell wall, and complete cell content (a). After 4 h of incubation with 0.75 mg/L chlorhexidine, treated cells showed detached cytoplasmic membrane from the cell wall at the two poles of a rod cell (arrows in b), leakage of cell content (arrow in c) and formation of ghost cells (d). These results were confirmed in two independent experiments. Magnification in all cases  = ×20,500. Bar  = 1 μm.

**Figure 7:**
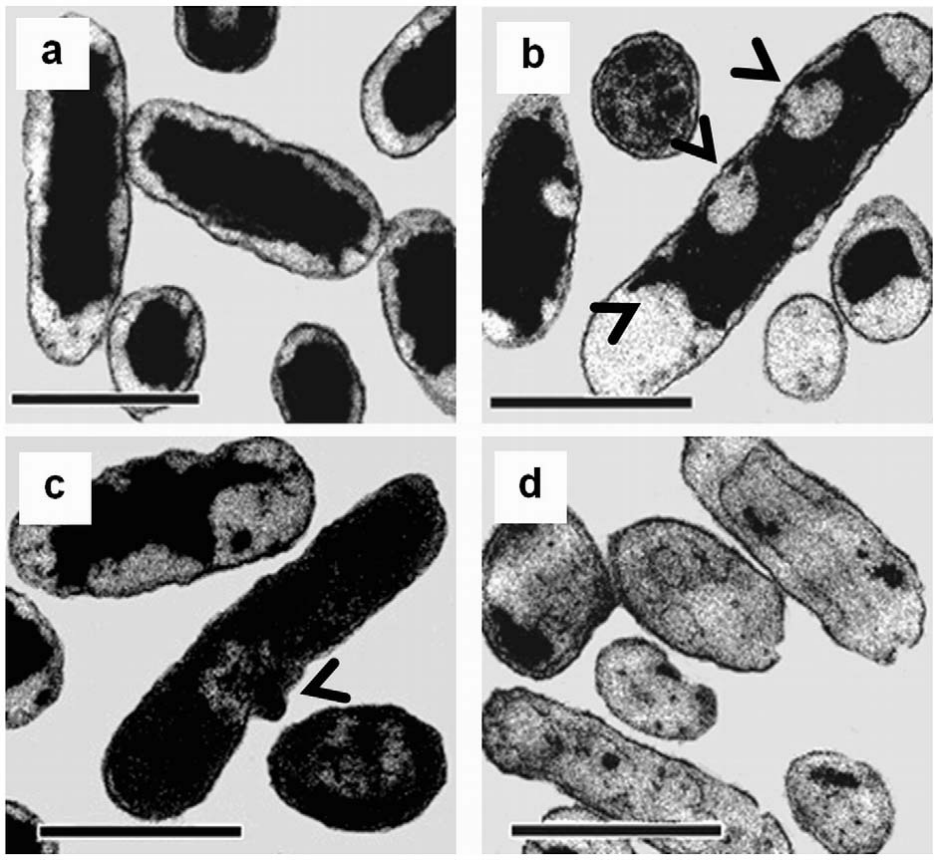
Cytological changes of *E. coli* after treated with chlorhexidine. Control cells possess intact cell membrane, cell wall, and complete cell content (a). After 4 h of incubation with 0.75 mg/L chlorhexidine, the treated *E. coli* cells showed detached cytoplasmic membrane from the cell wall at both poles and cylindrical part of the cells (see arrows in b), leakage of cell content (see arrow in c) and formation of ghost cells (d). These results were confirmed in two independent experiments. Magnification in all cases  = ×20,500. Bar  = 1 μm.

Although the scanning electron microscopy on the CHX-treated cells showed that it caused morphological changes on the cell surfaces of the bacteria, we also investigated whether the same concentration of CHX might affect the internal structure of these cells. We utilized transmission electron microscopy to reveal the changes occurred inside the CHX-treated cells. As expected, significant cytological changes were observed in both CHX-treated *B. subtilis* (Fig. 6) and *E. coli* cells (Fig. 7) when compared to the controls. In both cases, the outer membrane of *E. coli* and the cytoplasmic membrane of *B. subtilis* were detached (Fig. 6b and 7b, black arrows) and formed bulges (Fig. 6c and 7c, black arrows) on their cell walls. These observations suggest that the dented spots found in the CHX-treated cells may be probably due to the detachment of phospholipid membrane from the cell wall [14], [17], [18]. The damaged membrane eventually led to the formation of ghost cells in both species upon prolonged CHX treatment (Fig. 6d and 7d). These ghost cells, however, still possessed a cell wall but not an intact cytoplasmic membrane. It has been reported that at high concentration, perfusion of CHX into the cells caused precipitation of cellular content in the cytoplasm [19]. Our TEM photomicrographs showed that the cellular contents might leak out through the damaged sites on the cell wall (Fig. 6c and 7c).

### Relationship between CHX and cellular phospholipids

**Figure 8:**
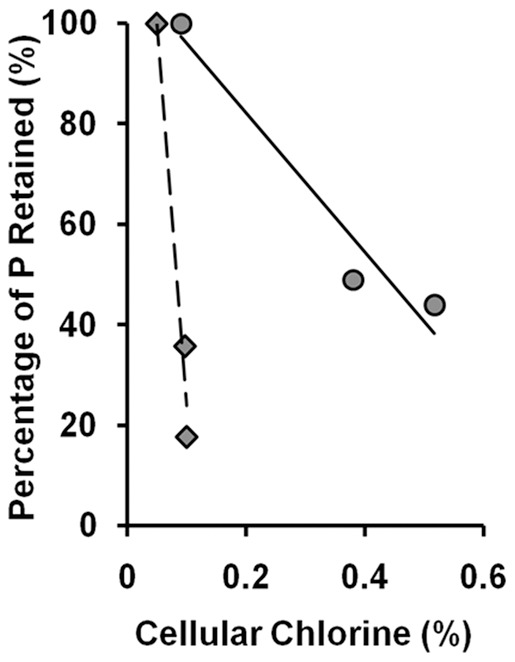
Effect of chlorhexidine exposure on phospholipids content in *B. subtilis* and *E. coli*. *B. subtilis* (diamond) and *E. coli* (circle) were treated with 0, 0.3, and 0.75 mg/L of chlorhexidine for 3 h and then subjected to EDAX to determine the contents of phosphorus and chlorine retained in the cells. The results were confirmed in two independent experiments.

There were reports showing that transmembrane disruptions could lead to losses of ions, ATP, and other metabolites from cellular content [17]. As suggested by Tattawasart et al. [14], chlorine (Cl) can represent the CHX taken up by the cells. Phosphorus (P), which is present in the phospholipid bilayers, the sugar-phosphate backbone of nucleic acids, and the nucleotide pool in the cytosol, could be used as an elemental marker to reflect damages on the cell wall and membranes. Energy dispersive X-ray analysis (EDAX) is a technique that can be used to determine the elemental composition in a small area observed under scanning electron microscope [12]. We utilized this technique to measure the cellular contents of Cl and P treated with 0, 0.3 and 0.75 mg/L of CHX. As shown in Fig. 8, there was an inverse relationship between the measured chlorine content, reflecting the uptakes of CHX, and the measured cellular phosphorus content, reflecting the phospholipids, nucleotide pool, and nucleic acids content in both *B. subtilis* and *E. coli* (diamonds and circles, respectively). With increasing concentration of chlorhexidine added to the culture, the percentage of P retained in *B. subtilis* dropped (Figure 8, diamonds) but the retention of Cl or CHX on the cell membrane was very little (less than 0.1%). In comparison, the Cl content or CHX retained in *E. coli* cells was higher than that of *B. subtilis* under the same concentration of CHX (circles). This experiment indicates that the leakage of cellular content (represented by P) was more severe in *B. subtilis* than *E. coli*, while the cell wall of *E. coli* retained more chlorhexidine (represented by Cl) than that of *B. subtilis*. The results from both the growth study and elemental analysis construed that *B. subtilis* was slightly more sensitive to CHX than *E. coli*, which may be attributed to their differences in their cell wall composition.

### Change in proteomes of the whole cell and cell wall of CHX-treated bacterial cells

**Figure 9:**
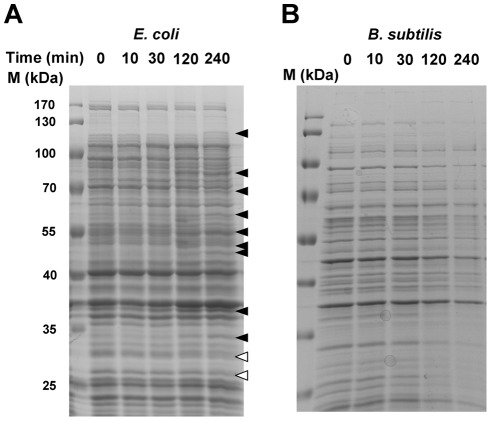
Time course of CHX treatment on the total protein contents in both species of bacteria. SDS polyacrylamide gel separation of the total cellular proteins from (A) *E. coli* and (B) *B. subtilis* samples treated with CHX at different time points. The black arrow heads indicate proteins that were induced by the CHX treatment and hollow arrow head indicates protein that was suppressed in expression after the CHX treatment. The experiments were repeated twice and one is shown here.

Based on the results of the above experiments, we inferred that the bacterial cell wall may have been damaged by CHX. We hypothesized that the cell wall integrity and its content may have been leached out of the cytoplasm and cell wall; therefore we investigated how the proteomes of the cell walls of both Gram-positive and -negative species were affected. CHX was added to the log phase cultures of both *E. coli* and *B. subtilis* and samples of them were removed at different time points. The total proteins from the samples were analyzed by SDS PAGE. In the experiment, we loaded equivalent number of bacteria by using the same optical density (OD) assuming that the OD is proportional to the concentration of the bacterial culture. Throughout the course of CHX treatment, the total protein content in *E. coli* was almost the same (Fig 9A), but there were some proteins induced (solid arrow head) and a few proteins suppressed or lost (open arrow). In contrast, in *Bacillus* the total amount of protein decreased with the time of treatment, suggesting that leaching of the cellular content occurred after CHX treatment (Fig 9B). During the 4 hours of CHX treatment, the optical densities of both *E. coli* and *B. subtilis* increased only 2.3-fold and 1.83 fold, respectively, indicating that the growth rate of both species were severely affected.

**Figure 10:**
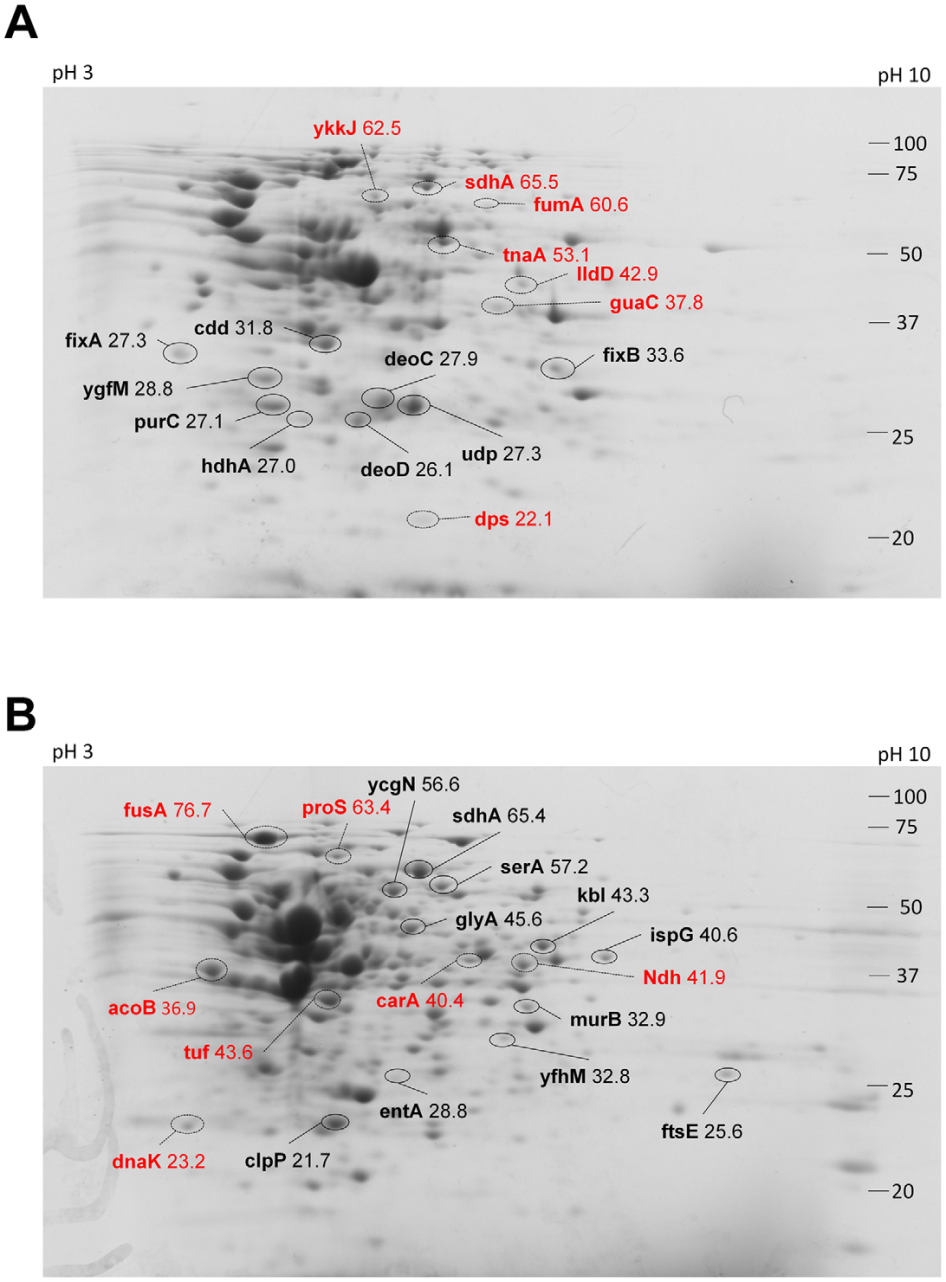
2D gel of cell wall/membrane proteins from *E. coli* and *B. subtilis* after CHX treatment. Proteins extracted from the cell wall fraction of (A) *E. coli* and (B) *B. subtilis* were separated by isoelectric focusing (pH 3–10) and subsequent 12% SDS polyacrylamide gel followed by Coomassie Blue staining. Protein spots that increased in intensity were circled with black solid lines and those with decreased in intensity were circled with broken lines. The numbers on the left were the the molecular weight of protein markers in kDa. The pI of the protein along the first dimension was shown on the top of the gel with pH 3 on the left and pH 10 on the right. The identity of the protein spots was labelled with their gene name and their molecular weight. The experiments were repeated twice and showed similar spot patterns.

Since we observed differential changes of proteins in both cell types, we then examined whether the proteins in their cell wall and membranes were also affected by CHX treatment. We isolated the proteins only from the cell wall/membrane of the bacterial lysates and then separated by 2D SDS polyacrylamide gel electrophoresis. Software analysis of the intensities of the protein spots between the treated and control samples identified proteins that were affected by CHX. In the 2D gel experiments, we loaded 150 μg of the purified cell wall/membrane proteins from each species onto an IEF stripe. In the case of *E. coli*, the comparison between the treated and untreated protein samples might infer the induction and repression of protein expression in the bacteria (Fig 10A). Around 30 proteins were flagged for changes in staining intensity above 1.4 fold compared with the control (untreated) sample. Among these proteins, 16 proteins were identified by MALDI-MS/MS (Table S1). A total of 9 proteins were up-regulated, which belong to enzymes involved in purine nucleoside, nucleotide conversion, and electron transport chain (Table 1). Seven proteins were found to be lower in expression in the cell wall of *E. coli*; however, most of the proteins were from different categories of function; for example, fumarate hydratase and flavoprotein subunit of succinate dehydrogenase are enzymes involved in the tricarboxylic acid (TCA) cycle, whereas lactate dehydrogenase is the enzyme used in anaerobic reduction of pyruvate. Even though the cell wall proteins were isolated using a traditional cell wall/membrane purification method [20], but only about half of these proteins were classified as membrane bound and while the other proteins were cytoplasmic, which were presumably extrinsic proteins associated tightly with the membrane.

**Table 1:** Summary of the proteins from the cell wall fractions of CHX-treated *E. coli* and *B. subtilis* identified by 2D gel and MALDI-MS/MS.

	Purine, nucleotide, nucleoside conversion (1)	Electron transport chain (2)	The Kreb cycle (3)	Amino acid metabolism (4)	Anaerobic respiration (5)	Stationary phase inducible (6)	Stress related protein (7)	Translation (8)	Others (9)	Total
*E. coli* increased	5	2							2	9
*E. coli* loss	1		2	1	1	1			1	7
*B. subtilis* increased				4			3		3	10
*B. subtilis* loss		1		2			1	3		7

Classification of the function of the identified proteins with the 2D mass spectrometry method was based on Gene Ontology from the UniProt Consortium. Each group of protein function is arbitrarily numbered in the bracket. The number (without the bracket) of identified protein classified according to their function is listed according to their increase or loss from the cell wall and the bacteria species.

For *B. subtilis*, the proteins involved were totally different and a majority of these proteins were cytoplasmic (Fig 10B, Table S1). Ten proteins of diverse functions were up-regulated after CHX treatment: 1-pyrroline-5-carboxylate dehydrogenase, 2-amino-3-ketobutyrate coenzyme A ligase, D-3-phosphoglycerate dehydrogenase, and serine hydroxymethyltransferase were enzymes involved in amino acid metabolism. Three stress-related proteins were also up-regulated, including: ATP-dependent Clp protease proteolytic subunit, putative hydrolase (yfhM), and 2,3-dihydroxybenzoate-2,3-dehydrogenase. Since the purification method used can isolate proteins associated with the cell walls and membranes of either the living cells or leaking cells with dented spots, the changes in protein may represent the responses of the cells to CHX. On the other hand, only 7 proteins were found to be down-regulated in cell wall fractions, and three of them were propyl-tRNA synthetase, elongation factor G and elongation factor Tu, which are involved in translation.

## Discussion

In this study, we found that CHX induces the formation of dented spots on the surface of the cell wall of both *E. coli* and *B. subtilis*. We argue that the formation of the dented spots was due to the action of CHX. First, very few dented spot were found in the absence of CHX, and second, the number of spots increased with the time of treatment and concentration of CHX. Surprisingly, the distribution of the dented spots along the cell body was not the same between the two species. In *B. subtilis*, the dented spots were preferentially located at the tip or cap region of the cell body, whereas in *E. coli* the spots were mainly found in the trunk. This suggests that the sites of action of CHX on *B. subtilis* and *E. coli* may be different. The morphological changes observed under transmission electron microscope on the two types of cells corroborate with those under scanning electron microscope. The frequency of finding collapses or dented spots in the cell wall around the cap region of *B. subtilis* was higher than that in the trunk region.

Based on the cell number as measured by the optical density of their culture, the total protein contents in the two species of bacteria were not the same during CHX treatment, and *B. subtilis* seems to be more susceptible to CHX as more proteins were lost from the cells (fig 9). Prolonged incubation with CHX, all cytoplasmic content was emptied to form ghost cells as visualized in the transmission electron photomicrographs, suggesting that CHX causes severe damages to the cell wall, including the cytoplasmic membrane and peptidoglycan layers in both species.

The energy-dispersive X-ray analysis (EDAX) on the CHX-treated bacterial cells demonstrated that the amounts of phospholipids, nucleic acids, and nucleotide pool (as the percent of phosphorus atom, P) retained in *B. subtilis* was less than that of *E. coli*. This indicates that the cell membrane of *B. subtilis* is more sensitive to CHX than that of *E. coli*. However, the steeper slope of the curve of the relative amount of P and Cl on *B. subtilis* shown in figure 8 only suggests that there is less chlorine atom (CHX) adsorbed onto the cell surface of *B. subtilis* than *E. coli*. It is possible that the negatively charged phosphorylated heptose and glucosamine moieties of the lipopolysaccharide (LPS)-containing cell wall of *E. coli* may lock up the more strongly cationic CHX molecules to make it less effective in function and this idea is consistent with the paradigm that the outer membrane of gram-negative bacteria acts as a permeability barrier to many cationic antibacterial agents [21]–[23].

The identification of 5 up-regulated proteins related to purine nucleotide and nucleoside conversion and 2 other proteins involved in the electron transport chain on the cell wall of *E. coli* were not expected, but a breach in the cellular integrity and content by CHX in the surviving bacterial cells may initiate a rescue mechanism of re-balance or maintenance of the cellular pool of nucleotides and proton gradient by enhancing the expression of these proteins in the cell wall. The levels of fumarate hydratase, flavoprotein subunit of succinate dehydrogenase, and lactate dehydrogenase were decreased, which may be due to a preferential loss from the cells during the treatment or suppression in expression due to an unknown reason. In contrast, there was a general loss of total proteins from *Bacillus* during CHX treatment, suggesting that CHX may breach the cell wall.

However, in the cell wall of *Bacillus subtilis* stress-related proteins and proteins involved in amino acid metabolism were up-regulated, and they may be used in responding to the stresses induced by CHX. However, we could identify only around 20 proteins out of the 40 protein spots differentially changed by CHX treatment. For a more comprehensive analysis of these proteins may require a larger scale of purification or another proteomic methods such as stable isotope labelling by amino acids in cell culture, SILAC, which do not rely on 2D gel separation and protein purification.

Disruption of the cell wall and cell membrane at a particular region along the body of bacteria by CHX may be a novel mechanism. CHX, a biguanide, exists in solution as a positively charged molecule, which can bind to anionic molecules on the cell wall. It is proposed that CHX kills bacteria by a disruption of membrane permeability rather than inhibition of ATPases on cell membrane [24]. It has been demonstrated that CHX destabilizes the outer membranes of Gram-negative bacteria to release proteins from periplasm but not the inner membrane [25] and decreased the lipid packing order of human buccal epithelial cell by fluorescence anisotropy study [26].

It is expected that the damages on the cell wall are evenly distributed if CHX acts on the LPS layer of the outer membrane in Gram-negative bacteria or teichoic acid on the peptidoglycan layer in Gram-positive bacteria [27], [28]. However, it has been demonstrated that the polar regions of *Bacillus* and *Lactobacillus* cell wall have a different composition of wall teichoic acid (WTA) from that of the trunk [29]. Sonnenfeld et al. showed that cationized ferritin (CF) binds specifically to the negatively charged groups in the cell poles of *B. subtilis*, suggesting that the cap cell wall surface is more electro-negative than that of the trunk [30]. Matsumoto et al. used cardiolipin-specific and phosphatidylethanolamine (PE)-specific fluorescent dyes to visualize the localization of the two lipid types in the membranes of *B. subtilis* and *E. coli* [31], [32]. Both cardiolipin and PE accumulate more specifically in the polar and septal regions in *B. subtilis*, whereas PE is more evenly distributed in *E. coli* [32]. It has also been shown that cardiolipin aggregates into domains in the cell membrane of *E. coli*, suggesting that the lipids in the cell membrane of bacteria may link in a raft-like structure as in mammalian cells. With the above observations and reports, we hypothesize that the structural changes of the bacterial cell wall may be due to the action of CHX on the cardiolipin-rich or PE-rich domains. PE molecules with a compact head-group can form a rigid network in the cell membrane, which makes it easier to form microdomain [33]. The localization of cardiolipin to the poles may be due to its interaction with peripheral membrane proteins to form patches on the membrane [34].

It is likely that CHX, containing both hydrophilic amine group and hydrophobic structure, can interact with the membrane cardiolipin and PE to disturb the normal arrangement and integrity of the phospholipid bilayer structure and its associated proteins. The disruption of proper lipid arrangement may lead to a collapse of the cell membrane and the formation of dented spots on the cell surface. And probably due to the uneven distribution of PE, cardiolipin, and other types of lipids in the cell membranes of *B. subtilis*, the poles of *B. subtilis* may be more susceptible to the damage caused by CHX.

It would be interesting to examine further whether other types of antimicrobials also cause the formation of dented spots specifically in *B. subtilis* and other Gram-positive bacteria. This can allow us to formulate a better hypothesis on how this type of antimicrobial kills bacteria.

## Supporting Information

Table S1 **A list of the proteins that were increased or decreased in quantity from the cell wall/membrane fraction of** ***E. coli*** **and** ***S. subtilis*** **after treated with CHX.** The gene names of the proteins were obtained from the UniProt Consortium. Fold-change was calculated by the intensity of the protein spot after the CHX treatment over that of the same protein before the treatment, and only those proteins with fold-change greater than 1.4 were displayed. Information on the localization and the functions of protein were obtained from the Biocyc database and the UniProt Consortium, respectively. The numbers on the category of the protein function were classified according to Table 1. The question mark represents unknown localization of the proteins in the cells.
(DOCX)

## References

[1] McBain AJ, Bartolo RG, Catrenich CE, Charbonneau D, Ledder RG (2003). Effects of a chlorhexidine gluconate-containing mouthwash on the vitality and antimicrobial susceptibility of *in vitro* oral bacterial ecosystems.. Appl Environ Microbiol.

[2] Sreenivasan PK, Gittins E (2004). The effects of a chlorhexidine mouthrinse on culturable microorganisms of the tongue and saliva.. Microbiol Res.

[3] Kanisavaran ZM (2008). Chlorhexidine gluconate in endodontics: an update review.. Int Dent J.

[4] Ellepola ANB, Samaranayake LP (2001). Adjunctive use of chlorhexidine in oval candidoses: a review.. Oral Dis.

[5] al-Tannir MA, Goodman HS (1994). A review of chlorhexidine and its use in special populations.. Spec Care Dentist.

[6] Bloomfield SF, Ascenzi JM (1996). Chlorhexidine and iodine formulations.. Handbook of Disinfectants and Antiseptics.

[7] Paulson DS (1993). Efficacy evaluation of a 4% chlorhexidine gluconate as a full-body shower wash.. Am J Infect Control.

[8] Glassman P (2003). Practical protocols for the prevention of dental disease in community settings for people with special needs: preface.. Spec Care Dentist.

[9] Longworth AR, Hugo WB (1971). Chlorhexidine.. Inhibition and destruction of the microbial cell.

[10] Teuber M (1973). Action of polymyxin B on bacterial membranes. II. Formation of lipophilic complexes with phosphatidic acid and phosphatidyl-glycerol.. Z Naturforsch.

[11] Khunkitti W, Hann AC, Lloyd D, Furr JR, Russell AD (1998). Biguanide-induced changes in *Acanthamoeba castellanii*: an electron microscopic study.. J Appl Microbiol.

[12] Hiom SJ, Hann AC, Furr JR, Russell AD (1995). X-ray microanalysis of chlorhexidine-treated cells of *Saccharomyces cerevisiae*.. Lett Appl Microbiol.

[13] Maillard JY, Hann AC, Beggs TS, Day MJ, Hudson RA, al et (1995). Energy dispersive analysis of X-rays study of the distribution of chlorhexidine diacetate and cetylpyridinium chloride on the *Pseudomonas aeruginosa* bacteriophage F116.. Lett Appl Microbiol.

[14] Tattawasart U, Hann AC, Maillard JY, Furr JR, Russell AD (2000). Cytological changes in chlorhexidine-resistant isolates of *Pseudomonas stutzeri*.. J Antimicrob Chemother.

[15] Cheung HY, Vitković L (1988). Coccus-shaped *Bacillus subtilis* cells are inhibited at stage 0 of sporulation.. Can J Microbiol.

[16] Shalamanov DS (2005). Chlorhexidine gluconate-induced morphological changes in gram negative microorganisms.. Biotechno & Biotechno Eq.

[17] Iwami Y, Schachtele CF, Yamada T (1995). Mechanism of inhibition of glycolysis in *Streptococcus mutans* NCIB 11723 by chlorhexidine.. Oral Microbiol Immunol.

[18] Steinberg D, Heling I, Daniel I, Ginsburg I (1999). Antibacterial synergistic effect of chlorhexidine and hydrogen peroxide against *Streptococcus sobrinus*, *Streptococcus faecalis* and *Staphylococcus aureus*.. J Oral Rehabil.

[19] Maris P (1995). Modes of action of disinfectants.. Rev Sci Tech.

[20] Wade HE, Robinson HK (1965). The distribution of ribosomal ribonucleic acids among subcellular fractions from bacteria and the adverse effect of the membrane fraction on the stability of ribosomes.. Biochem J.

[21] Nikaido H, Vaara M (1985). Molecular basis of bacterial outer membrane permeability.. Microbiol Rev.

[22] Hancock REW (1984). Alterations in outer membrane permeability. Annu Rev Microbiol..

[23] Stickler DJ, Thomas B, Clayton CL, Chawla JC (1983). Studies on the genetic basis of chlorhexidine resistance. Br J Clin Pract..

[24] Kuyyakanond T, Quesnel LB (1992). The mechanism of action of chlorhexidine.. FEMS Microbiol Lett.

[25] Barrett-Bee K, Newboult L, Edwards S (1994). The membrane destabilising action of the antibacterial agent chlorhexidine.. FEMS Microbiol Lett.

[26] Audus KL, Tavakoli-Saberi MR, Zheng H, Boyce EN (1992). Chlorhexidine effects on membrane lipid domains of human buccal epithelial cells.. J Dent Res.

[27] Doyle RJ, McDannel ML, Helman JR, Streips UN (1975). Distribution of teichoic acid in the cell wall of Bacillus subtilis.. J Bacteriol.

[28] Merad T, Archibald AR, Hancock IC, Harwood CR, Hobot JA (1989). Cell wall assembly in Bacillus subtilis: visualization of old and new wall material by electron microscopic examination of samples stained selectively for teichoic acid and teichuronic acid.. J Gen Microbiol.

[29] Andre G, Deghorain M, Bron PA, van Swam II, Kleerebezem M (2011). Fluorescence and atomic force microscopy imaging of wall teichoic acids in Lactobacillus plantarum.. ACS Chem Biol.

[30] Sonnenfeld EM, Beveridge TJ, Doyle RJ (1985). Discontinuity of charge on cell wall poles of Bacillus subtilis.. Can J Microbiol.

[31] Kawai F, Shoda M, Harashima R, Sadaie Y, Hara H (2004). Cardiolipin domains in Bacillus subtilis marburg membranes.. J Bacteriol.

[32] Nishibori A, Kusaka J, Hara H, Umeda M, Matsumoto K (2005). Phosphatidylethanolamine domains and localization of phospholipid synthases in *Bacillus subtilis* membranes.. J Bacteriol.

[33] Elder M, Hitchcock P, Mason FRS, Shipley GG (1977). A refinement analysis of crystallography of the phospholipid, 1,2-dilauroyl-DL-phosphatidylethanolamine, and some remarks on lipid–lipid and lipid–protein interactions.. Proc R Soc London A.

[34] van den Brink-van der Laan E, Boots JW, Spelbrink RE, Kool GM, Breukink E (2003). Membrane interaction of the glycosyltransferase MurG: a special role for cardiolipin.. J Bacteriol.

