# Supplementary material for: Differential Actions of Chlorhexidine on the Cell Wall of Bacillus subtilis and Escherichia coli
Source: PLoS One. 2012 May 11;7(5):e36659. doi: 10.1371/journal.pone.0036659 (PMC3350502; doi:10.1371/journal.pone.0036659)
Supplement: Table S1 — A list of the proteins that were increased or decreased in quantity from the cell wall/membrane fraction of E. coli and S. subtilis after treated with CHX. The gene names of the proteins were obtained from the UniProt Consortium. Fold-change was calculated by the intensity of the protein spot after the CHX treatment over that of the same protein before the treatment, and only those proteins with fold-change greater than 1.4 were displayed. Information on the localization and the functions of protein were obtained from the Biocyc database and the UniProt Consortium, respectively. The numbers on the category of the protein function were classified according to Table 1. The question mark represents unknown localization of the proteins in the cells. (DOCX) [file pone.0036659.s001.docx]

| ***E. coli increased*** | |  |  |  |  |
| --- | --- | --- | --- | --- | --- |
| **Gene name** | **Full name** | **Fold-change** | **Localization** | **Functions** | **Category** |
| **deoD** | purine nucleoside phosphorylase | 1.4 | Cytosol | purine nucleoside interconversion & catabolism | 1 |
| **cdd** | cytidine/deoxycytidine deaminase | 1.6 | cytosol | purine nucleoside interconversion | 1 |
| **udp** | uridine phosphorylase | 1.4 | cytosol | nucleoside & nucleotide interconversion | 1 |
| **purC** | phosphoribosylaminoimidazole-succinocarboxamide synthase | 2 | cytosol/ membrane | Biosynthesis of purine and certain amino acids | 1 |
| **deoC** | deoxyribose-phosphate aldolase | 4.7 | cytosol and membrane | involved in the catabolism of deoxyribonucleosides | 1 |
| **fixA** | predicted electron transfer flavoprotein subunit, ETFP adenine nucleotide-binding domain or protein FixA | 1.4 | Possible membrane bound | Flavoprotein subunit required for anaerobic carnitine metabolism; important in electron transport | 2 |
| **fixB** | electron transfer flavoprotein, alpha subunit or putative electron transfer flavoprotein FixB | 1.6 | membrane | Flavoprotein subunit required for anaerobic carnitine metabolism; important in electron transport | 2 |
| **ygfM** | putative selenate reductase subunit YgfM | 1.5 | Possible cytosolic | FAD binding unit for selenium reduction; UDP-N-acetylmuramate dehydrogenase activity | 9 |
| **hdhA** | 7-alpha-hydroxysteroid dehydrogenase | 1.6 | cytosol | Dehydroxylation of cholic acid (bile); sterol metabolism | 9 |

| ***E. coli* loss** | |  |  |  |  |
| --- | --- | --- | --- | --- | --- |
| **fumA** | fumarate hydratase (fumarase A), aerobic Class I | 1.8 | cytosol | TCA cycle | 3 |
| **sdhA** | succinate dehydrogenase flavoprotein subunit | 1.7 | Peripheral membrane protein; Cytoplasmic side | TCA cycle | 3 |
| **tnaA** | tryptophanase | 2.3 | Membrane | L-cysteine catabolism | 4 |
| **guaC** | GMP reductase or guanosine 5'-monophosphate oxidoreductase | 1.6 | Cytosol | G-> A nucleotide interconversion | 1 |
| **lldD** | L-lactate dehydrogenase, FMN-linked | 2 | membrane | Anaerobic respiration | 5 |
| **dps** | DNA starvation/stationary phase protection protein Dps | 1.4 | membrane | Nucleoid protein in stationary phase and iron acquisition | 6 |
| **ykkJ** | ABC transporter, ATP-binding protein | 1.4 | membrane | Transport protein | 9 |

| ***B. subtilis* increased** | |  |  |  |  |
| --- | --- | --- | --- | --- | --- |
| **clpP** | ATP-dependent Clp protease proteolytic subunit | 1.4 | Cytoplasmic | Stress response protein; a serine peptidase | 7 |
| **yfhM** | putative hydrolase | 2.4 | ? | Stress response protein induced by ethanol; a hydrolytic enzyme | 7 |
| **entA** | 2,3-dihydroxybenzoate-2,3-dehydrogenase | 2.8 | Cytoplasmic | Enterobactin biosynthesis, response to stress | 7 |
| **ycgN** | ycgN or 1-pyrroline-5-carboxylate dehydrogenase | 1.5 | Cytoplasmic | Proline biosynthesis; arginine degradation | 4 |
| **kbl** | 2-amino-3-ketobutyrate coenzyme A ligase | 1.7 | Cytoplasmic | Threonine degradation | 4 |
| **serA** | D-3-phosphoglycerate dehydrogenase | 1.7 | Cytoplasmic | L-serine biosynthesis | 4 |
| **glyA** | serine hydroxymethyltransferase | 1.8 | Cytoplasmic | Glycine/serine biosynthesis | 4 |
| **ispG** | 4-hydroxy-3-methylbut-2-en-1-yl diphosphate synthase | 1.7 | plasma membrane | isoprenoid biosynthesis | 9 |
| **murB** | UDP-N-acetylenolpyruvoylglucosamine reductase | 1.7 | Cytoplasmic | Cell wall/peptidoglycan biosynthesis | 9 |
| **ftsE** | cell division ATP-binding protein FtsE or cell-division ABC transporter (ATP-binding protein) | 2.1 | Membrane | Cell division transporter, sporulation | 9 |

| ***B. subtilis* loss** | |  |  |  |  |
| --- | --- | --- | --- | --- | --- |
| **proS** | prolyl-tRNA synthetase | 1.4 | Cytoplasmic | Protein translation | 8 |
| **fusA** | elongation factor G | 1.4 | Cytoplasmic | Translation elongation | 8 |
| **tuf** | elongation factor Tu | 2.8 | Cytoplasmic | Translation elongation | 8 |
| **acoB** | acetoin dehydrogenase E1 component (TPP-dependent beta subunit) | 1.4 | Membrane | acetoin biosynthesis or degradation into acetylCoA (Biosynthesis of branch chain amino acids) | 4 |
| **dnaK** | DnaK polypeptide | 1.4 | Cytoplasmic | protein folding, DNA replication, response to stress | 7 |
| **carA** | carbamoyl phosphate synthase small subunit | 1.7 | ? | Arginine biosynthesis | 4 |
| **ahpF** | NADH dehydrogenase | 1.4 | Membrane | Electron transport chain, oxidation-reduction reaction | 2 |

**Table S1** **A list of proteins that were increased or decreased from the cell wall/membrane of *E. coli* and *S. subtilis* after treated with CHX*.***

The gene name of the protein is obtained from the UniProt Consortium. Fold-change was calculated by the intensity of the protein spot after CHX treatment over that of the same protein before treatment, and only those proteins with fold-change greater than 1.4 were displayed. Information on the localization and functions of protein were obtained from the Biocyc database and the UniProt Consortium, respectively. The number on the category of the protein function was classified according to Table 1. The question mark represents unknown localization.
